# RETRACTION: Mechanism of p27 Upregulation Induced by Downregulation of Cathepsin B and Upar in Glioma

**DOI:** 10.1002/1878-0261.70288

**Published:** 2026-06-08

## Abstract

**RETRACTION:** S. Gopinath, K. Alapati, R. R. Malla, C. S. Gondi, S. Mohanam, D. H. Dinh, and J. S. Rao, “Mechanism of p27 Upregulation Induced by Downregulation of Cathepsin B and Upar in Glioma,” *Molecular Oncology 5*, no. 5 (2011): 426–437, https://doi.org/10.1016/j.molonc.2011.07.004.

The above article, published online on 26 July 2011 in Wiley Online Library (wileyonlinelibrary.com), has been retracted by agreement between the journal Editor in Chief, Kevin Ryan; the Federation of European Biochemical Societies; and John Wiley and Sons Ltd. Following publication, concerns were raised by a third party regarding the duplications of image elements Figure 7, as well as duplicated western blot bands between Figures 4C and 6B, between Figures 1C and 4C, within Figure 3A and within Figure 6A.

Internal investigation confirmed the duplications in these figures and additionally identified undeclared splice marks within Figures 2D and 4C, duplicated western blot bands between Figure 3A and Figure 4A of a previous publication with shared authors (DOI: 10.1371/journal.pone.0011668) where the same bands were presented in a different scientific context. The retraction has been agreed because the concerns were determined to affect the authenticity and reliability of the data and results presented. The authors have been informed of the retraction decision where possible; however, current contact details could not be verified.

**RETRACTION:** S. Gopinath, K. Alapati, R. R. Malla, C. S. Gondi, S. Mohanam, D. H. Dinh, and J. S. Rao, “Mechanism of p27 Upregulation Induced by Downregulation of Cathepsin B and Upar in Glioma,” *Molecular Oncology 5*, no. 5 (2011): 426–437, https://doi.org/10.1016/j.molonc.2011.07.004.

The above article, published online on 26 July 2011 in Wiley Online Library (wileyonlinelibrary.com), has been retracted by agreement between the journal Editor in Chief, Kevin Ryan; the Federation of European Biochemical Societies; and John Wiley and Sons Ltd. Following publication, concerns were raised by a third party regarding the duplications of image elements Figure 7, as well as duplicated western blot bands between Figures 4C and 6B, between Figures 1C and 4C, within Figure 3A and within Figure 6A.

Internal investigation confirmed the duplications in these figures and additionally identified undeclared splice marks within Figures 2D and 4C, duplicated western blot bands between Figure 3A and Figure 4A of a previous publication with shared authors (DOI: 10.1371/journal.pone.0011668) where the same bands were presented in a different scientific context. The retraction has been agreed because the concerns were determined to affect the authenticity and reliability of the data and results presented. The authors have been informed of the retraction decision where possible; however, current contact details could not be verified.

